# Supplementary material for: Applications of teledentistry in a French inmate population: A one-year observational study
Source: PLoS One. 2021 Apr 7;16(4):e0247778. doi: 10.1371/journal.pone.0247778 (PMC8026055; doi:10.1371/journal.pone.0247778)
Supplement: Attachment — Submitted filename: PONE-D-20-36871_reviewer 1.docx [file pone.0247778.s002.docx]

PLOS ONE

Applications of Teledentistry in a French Inmate Population: A one-year observational study

--Manuscript Draft--

| Manuscript Number: | PONE-D-20-36871 |
| --- | --- |
| Article Type: | Research Article |
| Full Title: | Applications of Teledentistry in a French Inmate Population: A one-year observational study |
| Short Title: | Teledentistry Inmate Population |
| Corresponding Author: | Sompop Bencharit, DDS, MS, PhD, FACP Virginia Commonwealth University Richmond, VA UNITED STATES |
| Keywords: | teledentistry; oral health; correctional; prisoners' health |
| Abstract: | Abstract:  An oral telemedicine device was evaluated for one year at the Villeneuve-lès- Maguelone Correctional Facility. The aim of the study was to simplify the obligatory dental consultation protocol at the entrance visit for new detainees. 1051 detainees were enrolled and 651 of them (58.9%) accepted an oral examination by telemedicine throughout the entire year of 2018. Only 1 inmate did not need treatment and 88.06% of those who have been examined had at least one untreated cavitated carious lesion. Forty-four percent of people who received a telemedicine check-up were referred to a dentist with a dental emergency. The use of oral telemedicine at the entry visit in a detention facility may facilitate the oral health screening without wasting the dentist's time, and may allow an optimization of the inmate's oral healthcare. |
| Order of Authors: | Camille Inquimbert |
|  | Ioan Balacianu |
|  | Nicolas Huyghe |
|  | Joao Pasdeloup |
|  | Paul Tramini |
|  | Fadi Meroueh |
|  | Sylvie Montal |
|  | Sompop Bencharit, DDS, MS, PhD, FACP |
|  | Nicolas Giraudeau |
| Additional Information: |  |
| Question | Response |
| Financial Disclosure  Enter a financial disclosure statement that describes the sources of funding for the work included in this submission. Review the [submission guidelines](http://journals.plos.org/plosone/s/submission-guidelines#loc-financial-disclosure-statement) for detailed requirements. View published research articles from [PLOS ONE](http://journals.plos.org/plosone/) for specific examples.  This statement is required for submission and will appear in the published article if | The authors received no specific funding for this work. |

*Powered by Editorial Manager® and ProduXion Manager® from Aries Systems Corporation*

| the submission is accepted. Please make sure it is accurate. |  |
| --- | --- |
| Unfunded studies  Enter: The author(s) received no specific funding for this work.  Funded studies  Enter a statement with the following details:   - Initials of the authors who received each award - Grant numbers awarded to each author - The full name of each funder - URL of each funder website - Did the sponsors or funders play any role in the study design, data collection and analysis, decision to publish, or preparation of the manuscript? - NO - Include this sentence at the end of your statement: The funders had no role in study design, data collection and analysis, decision to publish, or preparation of the manuscript. - YES - Specify the role(s) played. |  |
| * typeset |  |
| Competing Interests  Use the instructions below to enter a competing interest statement for this submission. On behalf of all authors, disclose any [competing interests](http://journals.plos.org/plosone/s/competing-interests) that could be perceived to bias this work—acknowledging all financial support and any other relevant financial or non- financial competing interests.  This statement will appear in the published article if the submission is accepted. Please make sure it is accurate. View published research articles from [PLOS ONE](http://journals.plos.org/plosone/) for specific examples. | The authors have declared that no competing interests exist. |

*Powered by Editorial Manager® and ProduXion Manager® from Aries Systems Corporation*

| NO authors have competing interests  Enter: The authors have declared that no competing interests exist.  Authors with competing interests  Enter competing interest details beginning with this statement:  I have read the journal's policy and the authors of this manuscript have the following competing interests: [insert competing interests here] |  |
| --- | --- |
| * typeset |  |
| Ethics Statement  Enter an ethics statement for this submission. This statement is required if the study involved:   - Human participants - Human specimens or tissue - Vertebrate animals or cephalopods - Vertebrate embryos or tissues - Field research   Write "N/A" if the submission does not require an ethics statement.  General guidance is provided below. Consult the [submission guidelines](http://journals.plos.org/plosone/s/submission-guidelines#loc-guidelines-for-specific-study-types) for detailed instructions. Make sure that all information entered here is included in the Methods section of the manuscript. | The study protocol was reviewed and approved by the Ministry of Social Affairs and Health, Decree approval no. AFSP1707688A. |

*Powered by Editorial Manager® and ProduXion Manager® from Aries Systems Corporation*

| Format for specific study types  Human Subject Research (involving human participants and/or tissue)   - Give the name of the institutional review board or ethics committee that approved the study - Include the approval number and/or a statement indicating approval of this research - Indicate the form of consent obtained (written/oral) or the reason that consent was not obtained (e.g. the data were analyzed anonymously)   Animal Research (involving vertebrate animals, embryos or tissues)   - Provide the name of the Institutional Animal   Care and Use Committee (IACUC) or other relevant ethics board that reviewed the study protocol, and indicate whether they approved this research or granted a formal waiver of ethical approval   - Include an approval number if one was obtained - If the study involved non-human primates, add additional details about animal welfare and steps taken to ameliorate suffering - If anesthesia, euthanasia, or any kind of animal sacrifice is part of the study, include briefly which substances and/or methods were applied   Field Research  Include the following details if this study involves the collection of plant, animal, or other materials from a natural setting:   - Field permit number - Name of the institution or relevant body that granted permission |  |
| --- | --- |
|  |  |
| Data Availability  Authors are required to make all data underlying the findings described fully available, without restriction, and from the time of publication. PLOS allows rare exceptions to address legal and ethical concerns. See the [PLOS Data Policy](http://journals.plos.org/plosone/s/data-availability) and [FAQ](http://journals.plos.org/plosone/s/data-availability#loc-faqs-for-data-policy) for detailed information. | Yes - all data are fully available without restriction |

*Powered by Editorial Manager® and ProduXion Manager® from Aries Systems Corporation*

| A Data Availability Statement describing where the data can be found is required at submission. Your answers to this question constitute the Data Availability Statement and will be published in the article, if accepted. |  |
| --- | --- |
| Important: Stating ‘data available on request from the author’ is not sufficient. If your data are only available upon request, select ‘No’ for the first question and explain your exceptional situation in the text box. |  |
| Do the authors confirm that all data underlying the findings described in their manuscript are fully available without restriction? |  |
| Describe where the data may be found in full sentences. If you are copying our sample text, replace any instances of XXX with the appropriate details.   - If the data are held or will be held in a public repository, include URLs, accession numbers or DOIs. If this information will only be available after acceptance, indicate this by ticking the box below. For example: All XXX files are available from the XXX database (accession number(s) XXX, XXX.). - If the data are all contained within the manuscript and/or Supporting Information files, enter the following: All relevant data are within the manuscript and its Supporting Information files. - If neither of these applies but you are able to provide details of access elsewhere, with or without limitations, please do so. For example:   Data cannot be shared publicly because of [XXX]. Data are available from the XXX Institutional Data Access / Ethics Committee (contact via XXX) for researchers who meet the criteria for access to confidential data.  The data underlying the results presented in the study are available from (include the name of the third party | N/A |

*Powered by Editorial Manager® and ProduXion Manager® from Aries Systems Corporation*

| and contact information or URL).   - This text is appropriate if the data are owned by a third party and authors do not have permission to share the data.   * typeset |  |
| --- | --- |
| Additional data availability information: |  |

*Powered by Editorial Manager® and ProduXion Manager® from Aries Systems Corporation*

[Click here to access/download;Manuscript;GiraudeauV3_11232020.docx](https://www.editorialmanager.com/pone/download.aspx?id=27868438&guid=67fde709-a4f7-49fc-9c5d-8c1b8ac4a9fb&scheme=1)

**Applications of Teledentistry in a French Inmate Population: A one-year observational study**

Camille Inquimbert^1,2^, Ioan Balacianu^3^, Nicolas Huyghe^2^, Joao Pasdeloup^2^, Paul Tramini^2,4^, Fadi Meroueh^3^, Sylvie Montal^2^, Sompop Bencharit^4^, Nicolas Giraudeau^2,5^

1 Systemic Health Care, EA 4129, University of Lyon 1, Lyon France

2 Dental department, University Hospital of Montpellier, Montpellier, France

3 Prison medical department, University Hospital of Montpellier, Montpellier, France ^4^Department of General Practice, School of Dentistry and Department of Biomedical Engineering, College of Engineering, Virginia Commonwealth University, Richmond, Virginia, USA

5 CEPEL, UMR 5112, University of Montpellier, CNRS, Montpellier, France

**Abstract:** (125 words)

An oral telemedicine device was evaluated for one year at the Villeneuve-lès-Maguelone Correctional Facility. The aim of the study was to simplify the obligatory dental consultation protocol at the entrance visit for new detainees. 1051 detainees were enrolled and 651 of them (58.9%) accepted an oral examination by telemedicine throughout the entire year of 2018. Only 1 inmate did not need treatment and 88.06% of those who have been examined had at least one untreated cavitated carious lesion. Forty-four percent of people who received a telemedicine check-up were referred to a dentist with a dental emergency. The use of oral telemedicine at the entry visit in a detention facility may facilitate the oral health screening without wasting the dentist's time, and may allow an optimization of the inmate's oral healthcare.

KEYWORDS: teledentistry, oral health, correctional, prisoners’ health

# INTRODUCTION:

In France, all new inmates entering a prison have the right of having an entry medical check- up (1). This consists of a visit with a physician, an interview with a psychologist, an interview with a nurse practitioner, and an oral check-up carried out by a dentist. Within each prison health unit, a medical team is not always available to carry out appropriate entry medical checkups in one visit. Therefore, the oral and dental examination is only carried out in 52% of cases and very often by a health professional who is not a dentist (2). This calls into question the quality of this entry check-up since only dentists are authorized and trained to make a dental assessment and proper dental diagnosis. Indeed, in France, the profession of second- class dental professionals such as dental hygienists in the US does not exist. There is a shortage of dentists to carry out entry medical check-ups. The Medical Department of the Villeneuve- lès-Maguelone Arrest House (VLM), managed by the University Hospital Center (CHU) of Montpellier therefore set up an oral telemedicine activity to fill in this gap. Initially, a trial experiment was carried out from 2015 to 2017 with funding from the Regional Health Agency (ARS) (3).

Telemedicine has been regulated in France since the telemedicine decree published in 2010 (4). Since then telemedicine has been deployed throughout the country in order to fight against the demographic inequality of public healthcare. Oral telemedicine in France was born in 2014 with the e-DENT project led by the Montpellier University Hospital, which set it up firstly for medico-social establishments hosting dependent elderly people and people with disabilities (5). In order to ensure the same quality of diagnosis by telemedicine as in face-to- face situations, an intraoral camera using fluorescence was chosen: the Soprocare® (Actéon Group, Mérignac, France). The study carried out by the Montpellier CHU showed a great similarity between the two techniques and validated the use of Soprocare® in the context of a telemedicine activity (6).

The inmate’s oral health environment was compared to those with low socioeconomic status. There is a real need for an improvement of the existing oral health care system exists. The oral health of detainees is usually very poor, as shown by various studies in France and around the world (7–9). This poor oral health may not only lead to general health problems, but also have a negative impact on the inmate's integration into his/her new environment in the prison as well as for his/her long-term reintegration into society and in the search for work after the prisoner is released.

The General Directorate of Health of the Ministry of Health launched a call for projects in 2017 to finance experiments aiming to improve oral dental consultations during the entrance visit. The Montpellier CHU responded and was selected for funding. The objective of the project was to offer all new entrants an oral check-up by telemedicine. The granted funding reached

€ 55,353 for the 2018 year. As part of this study, we therefore analyzed the data collected during this project. The main objective was to analyze the implementation of oral telemedicine in the VLM Health Unit during the 2018 year. The secondary objectives were to evaluate the caries experience and the urgency level, and to collect information about the patients’ practitioners.

# MATERIALS AND METHODS:

The study protocol was approved by the General Directorate of Health of the Ministry of Health. This was a cross-sectional observational study for a period of one year, from January 3, 2018 to December 31, 2018 conducting at the Sanitary Unit of the VLM remand center, Emergency Department of the Montpellier University Hospital. All new incomers were enrolled for the year of 2018 with the following inclusion and exclusion criteria (Table 1)

# Table 1 Inclusion and Exclusion Criteria

| **Inclusion criteria:** | **Exclusion criteria:** |
| --- | --- |
| Be a new member of the Villeneuve-lès- Maguelone remand center | Be in a mental state that does not allow the patient to understand the operator's instructions |
| Consent to benefit from an oral examination as part of the initial medical examination |  |

The oral telemedicine activity took place in a dedicated room with the following set up:

- an armchair so that the patient can sit down and lie down slightly,
- a stool with wheels so that the caregiver can sit facing the patient,
- a laptop computer on which is installed specific telemedicine software for collecting general information and videos,
- a pedal connected to the computer by a USB cable to start and stop the video captions,
- a Soprocare® intraoral camera connected to the computer by a USB cable.

A caregiver was equipped with examination gloves and a mask at the examination site with the new inmate, while a remote dentist was equipped with only a computer with an internet connection.

This activity was taking place asynchronously. The remote dentist was online but not waiting behind his computer for the patient and caregiver to be ready. A caregiver offered all new arrivals to receive an oral check-up by telemedicine. If they accepted, they were accompanied to the room dedicated to this activity. The procedure was explained to them in detail, including the fact that the data would be transferred safely to a secured server to allow analysis by a dental surgeon from the public health service of the CHU of Montpellier. Once the inmate had been informed and his consent had been obtained, the caregiver asked a number of following questions:

- Do you have a “referring” dentist?
- If so, can you give its name or the type of structure (mutual center, CHU, other US)?
- What is the date of the last visit to the dentist? (1 month, 6 months, 1 year, 5 years, 10 years and more)

Then, the caregiver completed an odontogram for each inmate on the software by identifying the missing teeth on a pre-filled dental diagram. Finally, a video of each dental quadrant was produced. The caregiver recorded all the surfaces of all the teeth with the 2 fluorescence modes. Any comments from the patient or the caregiver can be directed to the remote dentist. Once all the information was collected, it was sent securely to the server. The remote dentist analyzed them and identified the teeth that needed to be treated, already treated, and absent. The DMFT score was then obtained. The medical team then was informed about the inmate’s oral-dental condition. An urgency score based on a validated classification (10) was appraised. The report was sent back through the system and integrated into the inmate's file. Depending on the emergency score, an appropriate referral and a dental appointment if needed was then made. Since none of the indices often followed a normal distribution, a non- parametric Mann-Whitney test was used to compare quantitative variables among different groups. Statistical analysis was performed using Stata v16.1 software (Statacorp, Texas, United States)

# RESULTS:

In 2018, 1,051 men entered VLM's remand center. 619 (58.90%) agreed to have an oral examination by telemedicine. The recording of the data was done mostly by a caregiver with some consultation with the remote dentist. The dentist for the prison could continue to treat patients all day without any interruption. The mean time to record data per inmate was 14 minutes and the mean time to analyse data was 8 minutes.

There were several reasons for inmates refusing the oral examination by telemedicine as listed below.

- Refusal of all or part of the entry medical examination
- Conditions making the assessment difficult or impossible such severe gag reflect
- Refusal of benefit from dental care
- Refusal of telemedicine
- Fear of dentists

Out of the 619 people which have been seen, 52 were edentulous and had removable complete denture prostheses and therefore no telemedicine assessment was therefore performed. The remaining 567 inmates received a telemedicine dental examination. The new inmate population were in the months of April, June, September and November, with the highest percentage of examinations in August. (Table 2)

# Table 2 Numbers of new inmates and dental telemedicine examinations

| **Months** | **Jan** | **Feb** | **Mar** | **Apr** | **May** | **Jun** | **Jul** | **Aug** | **Sep** | **Oct** | **Nov** | **Dec** | **TOTAL** |
| --- | --- | --- | --- | --- | --- | --- | --- | --- | --- | --- | --- | --- | --- |
| **New comers** | 76 | 89 | 75 | 101 | 87 | 100 | 89 | 71 | 102 | 78 | 103 | 80 | **1051** |
| **Visit** | 44 | 49 | 43 | 51 | 48 | 59 | 53 | 57 | 63 | 42 | 55 | 55 | **619** |
| **Refusal** | 32 | 40 | 32 | 50 | 39 | 41 | 36 | 14 | 39 | 34 | 48 | 25 | **430** |
| **Prosthesis** | 5 | 6 | 0 | 8 | 5 | 8 | 4 | 3 | 3 | 2 | 4 | 4 | **52** |
| **% examined** | **58%** | **55%** | **57%** | **50%** | **55%** | **59%** | **60%** | **80%** | **62%** | **54%** | **53%** | **69%** | **58.9%** |

The mean age was 31.65+/-10.69 years and a minimum age of 16 and maximum of 76 years. Out of the 567 patients who underwent the telemedicine examination, we were only able to obtain an accurate DMFT for 561 of them only.

Specific to the question: "Do you have a treating dentist?” the answers were distributed in Table 3. Majority of inmates, 51.68%, 293, answered no.

# Table 3 Numbers of inmates in regards to having a dentist

| Treating dentist | | | | |
| --- | --- | --- | --- | --- |
| NO |  | 406 | 7.37% | 72.37% |
| YES | Doesn’t know the  name | 99 | 17.65% | 27.63% |
|  | Knows the name | 50 | 8.91% |  |
|  | Others (CSERD,  mutual, other US) | 6 | 1.07% |  |
|  |  | 561 |  |  |

In regards to the question, “What was the date of your last visit to the dentist?” The average answer was 30.87+/- 36.78 month or just about 2 and a half years (Table 4).

# Table 4 Reported last time seeing a dentist

| Last visit to the dentist | | |
| --- | --- | --- |
| 1 month | 65 | 11,46% |
| 6 months | 96 | 16,93% |
| 12 months | 225 | 39,68% |
| 60 months | 117 | 20,63% |
| 120 months | 64 | 11,29% |
|  | 567 |  |

**Table 5 DMFT distribution**

|  | *n* | *%* |
| --- | --- | --- |
| no decayed tooth | 68 | 12.12% |
| at least one decayed teeth | 493 | 87.88% |
| no decayed filled tooth | 368 | 65.60% |
| at least one decayed filled teeth | 193 | 34.40% |
| no extraction needed | 518 | 92.30% |
| at least one extraction needed | 43 | 7.70% |
| no filled tooth | 302 | 53.83% |
| at least one filled tooth | 259 | 46.17% |
| no missing tooth | 461 | 82.17% |
| at least one missing tooth | 100 | 17.83% |
| DMFT=0 | 52 | 9.27% |
| DMFT>0 | 509 | 90.73% |

**Figure 1 Histogram of DMFT distributions**

The figure 1 and the table 4 demonstrate the distribution of the DMFT score in this population of detainees, with a mean value of 7.76 (+/- 5.31) and 9.27% caries-free. The mean components of DMFT were also assessed:

D = 5.78 (+/- 4.64), M = 0.29 (+/- 0.82), F = 1.68 (+/- 2.93).

The mean number of cavitated lesions per inmate was 2.93 (3.11), and the mean number of enamel lesions was 0.87 (1.49). The mean number filled teeth with cavitated lesions was 0.74 (1.39).

# Table 6 Distribution of emergency score

Urgency score:

The average urgency score was 2.76 +/- 1.13

| Urgency score | | |
| --- | --- | --- |
| 0: no need for care | 1 | 0.18% |
| 1: need for a scaling | 59 | 10.41% |
| 2: low emergency | 231 | 40.74% |
| 3: advanced urgency | 14 | 2.47% |
| 4: high urgency | 234 | 41.27% |
| Undetermined | 28 | 4.94% |
|  | 567 | 100.00% |

# Table 7 Distribution of treatment after teledentistry

| **Month** | **Jan** | **Feb** | **Mar** | **Apr** | **May** | **Jun** | **Jul** | **Aug** | **Sep** | **Oct** | **Nov** | **Dec** | **TOTAL** |
| --- | --- | --- | --- | --- | --- | --- | --- | --- | --- | --- | --- | --- | --- |
| **New comers** | 76 | 89 | 75 | 101 | 87 | 100 | 89 | 71 | 102 | 78 | 103 | 80 | **1051** |
| **Visit** | 44 | 49 | 43 | 51 | 48 | 59 | 53 | 57 | 63 | 42 | 55 | 55 | **619** |
| **Refusal** | 32 | 40 | 32 | 50 | 39 | 41 | 36 | 14 | 39 | 34 | 48 | 25 | **430** |
| **Prosthesis** | 5 | 6 | 0 | 8 | 5 | 8 | 4 | 3 | 3 | 2 | 4 | 4 | **52** |
| **% examined** | **58%** | **55%** | **57%** | **50%** | **55%** | **59%** | **60%** | **80%** | **62%** | **54%** | **53%** | **69%** | **58.9%** |
| **Appointment after**  **teledentistry** | 19 | 19 | 16 | 22 | 26 | 26 | 28 | 22 | 25 | 20 | 12 | 15 | **250** |
| **% / new comers** | **25%** | **21%** | **21%** | **22%** | **30%** | **26%** | **31%** | **31%** | **25%** | **26%** | **12%** | **19%** | **24%** |
| **% / visits** | **43%** | **39%** | **37%** | **43%** | **54%** | **44%** | **53%** | **39%** | **40%** | **48%** | **22%** | **27%** | **40%** |

**Table 8 Mean score of emergency crossed by no practitioner and practitioner groups**

The mean urgency score was not significantly different when individuals had a regular practitioner or not.

|  | **score of emergency** | | |
| --- | --- | --- | --- |
|  | *mean* | *sd* | *n* |
| **No practitioner** | 2.76 | 1.15 | 385 (72.4%) |
| **Regular practitioner** | 2.77 | 1.05 | 147 (27.6%) |
| Mann-Whitney test | 0.89 |  | |

# Table 9 Mean DMFT score crossed by no practitioner and practitioner groups

The mean DMFT score was significantly different when individuals had a regular practitioner or not.

|  | **DMFT** | | |
| --- | --- | --- | --- |
|  | *mean* | *sd* | *n* |
| **No practitioner** | 7.37 | 5.26 | 406 (72.5%) |
| **Regular practitioner** | 8.75 | 5.33 | 155 (27.5%) |
| Mann-Whitney test | 0.005 |  | |

**Figure 2 Mean DMFT score for groups with no and with practitioner**

# Table 10 Mean last appointment period crossed with with no practitioner and with practitioner groups

The mean last appointment time was significantly different when individuals had a regular practitioner or not.

|  | **last appointment** | | |
| --- | --- | --- | --- |
|  | *mean* | *sd* | *n* |
| **No practitioner** | 36.71 | 39.04 | 406 (72.5%) |
| **Regular practitioner** | 14.91 | 22.75 | 155 (27.5%) |
| Mann-Whitney test | 0.0001 |  | |

**Table 11 Mean extractions needed crossed by no practitioner and practitioner groups** The mean extractions needed was not significantly different when individuals had a regular practitioner or not.

|  | **extractions needed** | | |
| --- | --- | --- | --- |
|  | *mean* | *sd* | *n* |
| **No practitioner** | 0.14 | 0.65 | 406 (72.5%) |
| **Regular practitioner** | 0.16 | 0.73 | 155 (27.5%) |
| Mann-Whitney test | 0.89 |  | |

# DISCUSSION:

Telemedicine, a functional and facilitating tool for the initial consultation on entry into detention and the organization of care:

Thanks to the oral telemedicine activity set up for one year, the service was able to offer all new arrivals an oral examination. Nationally, initial oral exam check-up for new inmates is only offered for 52% of all inmates (11). Previously, it had never been possible to find any organization in France that would allow this initiative. It is important to note that the use of telemedicine allows the diagnosis to be made by an oral health expert, a dentist, not by other health professionals who may not be competent in this discipline.

More importantly, Teledentistry allows the dentist to concentrate on the patient care and treatment appointments that he has planned throughout the day and thus continue smoothly his activity without being impacted by the entrance examinations. Because the number of new inmate arrivals are often not known, without our screening teledentistry, a dentist may have to stand by doing nothing all day waiting for the inmate arrival. Indeed, the number of inmate arrivals can vary depending on the judicial activity and can be unlimited. Telemedicine makes it possible to avoid having to summon the detainee again after the arrival and also avoid unnecessary overcrowding in the dental department.

In our study, the images were reviewed by a practitioner in the dental department of the University Hospital of Montpellier, but the data analysis can also easily be done by the dentist working in the prison. The advantage of asynchronous organization is that the prison dentist can analyze the data at the end of his day or when he has a moment in his working day (in the absence of a patient, or when he completed treatment prior to the plan) thus he optimizes his working time. Our protocol allows the same practitioner who analyzes the data to later provide care to the inmate if indicated afterwards. However, as we did in our study, an

agreement between the remote practitioner who analyzes and the one who will provide the care is very easy to set up.

As we can see in Table 7 XX, 40% of the patients who benefited from a telemedicine examination were quickly arranged for indicated treatment. These 40% are made of the patients with an emergency score equal to 4 identified in Table 6. This prioritization of care was made possible thanks to telemedicine and allowed people who needed it most to be taken care of first. In addition, telemedicine diagnosis makes it possible to request a panoramic X- ray prior to the dentist's appointment and thus save treatment time.

This telemedicine activity has started gradually in the service since 2013 (3) but has only been offered in respect of this process since January 2018. Since then, this organization has continued and the number of refusals to the oral examination by telemedicine decreased. The use of oral telemedicine demanded a change in the detainees’ management organization during the entrance visit; this sometimes took a little time, in order to change everyone's habits but today all people working in this department are delighted with the tool and process implementation. The doctor and / or nurses can learn about the inmates' oral health without disturbing the dentist since the oral telemedicine report is integrated into the patient's computerized medical record.

A very significant need for oral care in a population without a real care pathway outside the prison:

Our study showed an average CAD index of 7.76 (+/- 5.31) with a very large average number of decayed teeth at 5.78 (+/- 4.63), a number of missing teeth smaller than 1 (0.29 on average) and a low number of treated teeth. These figures are a little lower than in the latest studies identified in the literature. In Kosovo (8), the average DMFT was 8.44 in 2018 and was 10.6 in Sweden in 2018 (9). The mean age of the male prison population is similar in the different studies. In our study, it reached 31.65 years of age. This relatively young age explains the low number of missing teeth. Indeed, only 17.83% (100) have at least one missing tooth. But 7.70%

(43) need tooth extraction which may increase the number of missing teeth. No including total-denture-prosthesis wearers in this study also may lower the mean DMFT score.

The oral health status was generally very poor among prisoners. We identified only 9.27% (52) with a DMFT equal to 0 and only 12.12% (68) did not present any decay. The need for treatment also reflects poor oral health. 87.88% (493) had at least one decayed tooth in their mouth. 34.40% (193) showed a treated tooth with recovery of decay and only 46.17% (259) had at least one treated tooth.

The lack of care and the exit from the health system are also reflected in the identification of a treating dentist by the detainee. Indeed, only 27.5% (155) declared having a referring practitioner. But it's interesting to refine this point with the fact that only 8.91% (50) knew the name of their dentist. This may reflect a lack of involvement in their oral health care. This confirms the average time of the last dentist appointment which was 2 and a half years. In a population with so many dental problems, this duration is far too long. The cross statistics show a correlation between the time spent since the last appointment and the identification of a practitioner. On the other hand, the study of the cross between the average DMFT on populations with a practitioner and those without any is interesting. The average DMFT among those who report having a practitioner is significantly greater when compared to those

who do not report a treating practitioner. This can be explained by the fact that this population consults a lot on an emergency basis and that there is therefore very often a treatment that is carried out during the visit. Few members of this population have to go to the dentist for a check-up. The emergency score shows that 84.48% (479) of them needed care. The average score was 2.76 correlated with what inmates declared about having an outside the prison practitioner or not.

# CONCLUSION:

The development of oral telemedicine is more and more under the spotlight and on the agenda as the World Health Organization has made it one of the modules of its mOralHealth program(12). The prison population is a unique population which requires a special consideration and oral health care. The time spent in prison could be used to wisely "upgrade" the inmate’s oral health and thus later facilitate their reintegration into society once the sentence has been served. The identification of oral pathologies upon entry into detention enables the prioritization and the organization of care. Telemedicine can be a real ally in achieving this goal.

Telemedicine is increasingly developing and the COVID-19 crisis that the world is experiencing is encouraging its use even further. This remote medical practice must be developed intelligently and in close collaboration and embark only the main objective of improving care and particularly of aiming to reduce inequalities in access to care. Telemedicine should not seek to replace a well-functioning organization that meets the needs of a population and / or a territory. Since there is a real need for oral health care in the inmate population, telemedicine appears to have a great value. It allows a systematization of the oral examination at the entry into custody by a competent professional. It further optimizes the time of each actor for the direct benefit of the patient who is in great need of care.

# References:

1. Ministère du travail, de l’emploi et de la santé, secrétariat d’état à la santé. Instruction DGS/MC1/DGOS/R4 n°2011-206 du 29 août 2011 relative à la réalisation d’un examen bucco- dentaire des personnes détenues lors de leur arrivée en établissement pénitentiaire et à la réduction du risque infectieux associé aux soins dentaires. août 29, 2011 p. 22.
2. Cour des comptes. Rapport Public annuel 2014 - Tome 1. Paris: Cour des Comptes; 2014 févr p. 480.
3. Giraudeau N, Inquimbert C, Delafoy R, Tramini P, Valcarcel J, Meroueh F. Teledentistry, new oral care tool for prisoners. International Journal of Prisoner Health. 12 juin 2017;13(2):124‑34.
4. Ministère de la santé et des sports. Décret n°2010-1229 du 19 octobre 2010 relatif à la télémédecine. Code de la santé publique, SASH1011044D oct 19, 2010 p. 3.
5. Giraudeau N, Valcarcel J, Tassery H, Levallois B, Cuisinier F, Tramini P, et al. Projet e- DENT : téléconsultation bucco-dentaire en EHPAD. European Research in Telemedicine / La Recherche Européenne en Télémédecine. juin 2014;3(2):51‑6.
6. Inquimbert C, Hirata-Tsuchiya S, Yoshii S, Molinari N, Nogue E, Roy C, et al. Concordance study between regular face-to-face dental diagnosis and dental telediagnosis using fluorescence. J Telemed Telecare. 5 janv 2020;1357633X1989411.
7. Haute Autorité de Santé. Stratégies de prévention de la carie dentaire [Internet]. Paris: HAS; 2010 mars p. 180. Disponible sur: https://www.has- sante.fr/portail/jcms/c_991247/fr/strategies-de-prevention-de-la-carie-dentaire
8. Zajmi L, Begzati A, Sejdini M, Berisha N, Krasniqi L. Oral Health of Lipjan Convicts: Kosovo Prison House. International Journal of Dentistry. 2018;2018:1‑7.
9. Priwe C, Carlsson P. Oral health status of male Swedish citizens at admission to prison. J Correct Health Care. oct 2018;24(4):382‑94.
10. Estupinan-Day S. Promoting oral health.
11. Ministère de la santé et des sports, Ministère de la justice et des libertés. Plan d’actions stratégiques 2010-2014 : politique de santé pour les personnes placées sous main de justice [Internet]. Paris; 2010 janv p. 86. Disponible sur: https://solidarites- sante.gouv.fr/IMG/pdf/plan_strategique_2010_2013_prise_en_charge_personnes_placees_ sous_main_de_justice.pdf
12. World Health Organization, International Telecommunication Union. Report of the Global mOralHealth Workshop, 10-12 October 2018, Montpellier, France. Geneva, Switzerland: World Health Organization (WHO/NMH/PND/2019.6); 2019 p. 35.
