# Supplementary material for: Applications of teledentistry in a French inmate population: A one-year observational study
Source: PLoS One. 2021 Apr 7;16(4):e0247778. doi: 10.1371/journal.pone.0247778 (PMC8026055; doi:10.1371/journal.pone.0247778)
Supplement: Attachment — Submitted filename: Responses_to_Review_R2_02082021.docx [file pone.0247778.s004.docx]

**RESPONSES TO COMMENTS FROM EDITOR/REVIEWERS**

**Introduction**: Last line of Introduction should be revised to say Patient's Primary care dentist instead of Patient's Practioner to be consistent with terminology used in other sections of manuscript.

RESPONSE: Appreciate the comment.

TEXT CHANGE: The sentence is now read: “... patient’s primary care dentist.’

**Results:** Please rephrase Line 10 of results, it is hard to read and needs grammatical correction. Sentence starting. This included refusal of ………….fear of dentists.

RESPONSE: We appreciate the comment.

TEXT CHANGE: The sentence is now read: **“**This included refusal of any medical exam, refusal of dental care or teledentistry, limited mouth opening or gag reflex, and fear of dentists.”

**Tables**: Please check formatting on table 3,6 and 8 so two or 3 digit numbers displayed are in one line.

RESPONSE: We appreciate the comment.

TEXT CHANGE: Changes have been made.

Please complete missing column lables in Tables 5 and 7. ( Currently missing n and % label)

RESPONSE: We appreciate the comment.

TEXT CHANGE: Changes have been made.

**References:** Please translate references 1,2,4,5,7,11 to English.

RESPONSE: We appreciate the comment.

TEXT CHANGE: Changes have been made.

Reference 10 is incomplete, please submit complete reference with online access date if its a webpage.

RESPONSE: We appreciate the comment.

TEXT CHANGE: Changes have been made.
